# Glutamatergic Neurons in Rodent Models Respond to Nanoscale Particulate Urban Air Pollutants *in Vivo* and *in Vitro*

**DOI:** 10.1289/ehp.1002973

**Published:** 2011-04-07

**Authors:** Todd E. Morgan, David A. Davis, Nahoko Iwata, Jeremy A. Tanner, David Snyder, Zhi Ning, Winnie Kam, Yu-Tien Hsu, Jeremy W. Winkler, Jiu-Chiuan Chen, Nicos A. Petasis, Michel Baudry, Constantinos Sioutas, Caleb E. Finch

**Affiliations:** 1 Davis School of Gerontology; 2 USC College; 3 Viterbi School of Engineering; 4 Department of Neurobiology; 5 Department of Chemistry, and; 6 Keck School of Medicine, University of Southern California, Los Angeles, California, USA

**Keywords:** air pollution, cultured cells, free radical, glia, glutamate receptor, inhalation, nanoscale particulate air pollutants, neuroinflammation, neuron, neurotoxicity

## Abstract

Background: Inhalation of airborne particulate matter (PM) derived from urban traffic is associated with pathology in the arteries, heart, and lung; effects on brain are also indicated but are less documented.

Objective: We evaluated rodent brain responses to urban nanoscale (< 200 nm) PM (nPM).

Methods: Ambient nPM collected near an urban freeway was transferred to aqueous suspension and reaerosolized for 10-week inhalation exposure of mice or directly applied to rat brain cell cultures.

Results: Free radicals were detected by electron paramagnetic resonance in the nPM 30 days after initial collection. Chronic inhalation of reaerosolized nPM altered selected neuronal and glial activities in mice. The neuronal glutamate receptor subunit (GluA1) was decreased in hippocampus, whereas glia were activated and inflammatory cytokines were induced [interleukin-1α (IL-1α), tumor necrosis factor-α (TNFα)] in cerebral cortex. Two *in vitro* models showed effects of nPM suspensions within 24–48 hr of exposure that involved glutamatergic functions. In hippocampal slice cultures, nPM increased the neurotoxicity of NMDA (*N*-methyl-d-aspartic acid), a glutamatergic agonist, which was in turn blocked by the NMDA antagonist AP5 [(2*R*)-amino-5-phosphonopentanoate]. In embryonic neuron cultures, nPM impaired neurite outgrowth, also blocked by AP5. Induction of IL-1α and TNFα in mixed glia cultures required higher nPM concentrations than did neuronal effects. Because conditioned media from nPM-exposed glia also impaired outgrowth of embryonic neurites, nPM can act indirectly, as well as directly, on neurons *in vitro*.

Conclusions: nPM can affect embryonic and adult neurons through glutamatergic mechanisms. The interactions of nPM with glutamatergic neuronal functions suggest that cerebral ischemia, which involves glutamatergic excitotoxicity, could be exacerbated by nPM.

In urban environments, exposure to airborne particulate matter (PM) derived from vehicular traffic has been associated with a variety of cardiovascular and respiratory outcomes (Araujo and Nel 2009; Iannuzzi et al. 2010; Künzli et al. 2010), for example, increased incidence of asthma (McConnell et al. 2010), impaired lung growth (Gauderman et al. 2007), and accelerated atherosclerosis (carotid wall thickening) (Künzli et al. 2010).

Epidemiological evidence suggests that cognitive functions are vulnerable to airborne pollutant exposure across the life span. In older women, low cognitive function was associated with proximity to vehicular traffic (Ranft et al. 2009), whereas in middle-age adults, lower cognitive performance was associated with exposure to ozone (Chen and Schwartz 2009). Cognitive impairments of school-age children have been associated with exposure to black carbon (BC), a marker for vehicle-derived PM (Suglia et al. 2008). Also, a prospective study of children showed associations of lower IQ with prenatal exposure to vehicle-derived polycyclic aromatic hydrocarbons (PAHs) (Perera et al. 2009), and autism prevalence was associated with residential proximity at birth to urban freeways (Volk et al. 2010). Rodent models also show varying memory impairment after exposure to ozone (Avila-Costa et al. 1999; Dorado-Martinez et al. 2001; Guerrero et al. 1999; Rivas-Arancibia et al. 2010) and vehicle-derived air pollutants (Zanchi et al. 2010).

Neuropathological findings consistent with these cognitive changes have been reported based on comparisons of samples collected from Mexico City and control cities (Tlaxcala and Veracruz), which represent extremes in ambient PM. Brains of children and young adult accident victims showed a higher prevalence of diffuse amyloid-β peptide deposits, activated glia, and oxidized DNA in residents of highly polluted Mexico City compared with subjects from control cites (Calderón-Garcidueñas et al. 2008). Canine brains from Mexico City also showed more extensive deposits of amyloid-β in cerebral arteries and pyramidal neurons, together with markers of inflammation and glial activation, than did the brains of canines from control cities (Calderón-Garcidueñas et al. 2003).

These findings extend to laboratory rodents, which differ importantly from canines as a model for human brain inflammatory responses. The presence of amyloid-β deposits in aging humans and canines adds complexity to environmental responses, because endogenous amyloid itself stimulates local inflammatory responses (Finch and Morgan 2007). The endogenous amyloid-β protein of rodents differs from that of humans and canines in three amino acid residues that render the rodent amyloid-β protein less neurotoxic. Thus, rodent models for brain responses to environmental agents resolve an amyloid-independent phase of inflammatory responses. Inhalation by mice of ambient vehicle-derived PM from an urban freeway for 2–6 weeks induced glial responses with elevations of interleukin-1α (IL-1α), tumor necrosis factor-α (TNFα), nuclear factor κB, and several protein kinases (Campbell et al. 2005 2009; Kleinman et al. 2008). These inflammatory changes, together with increased lipid oxidation (Zanchi et al. 2010), suggest that chronic inhalation of vehicle- derived PM can cause inflammation and oxidative stress in the brain.

Although the bioactivities of airborne PM are incompletely understood, it is clear that the inhaled particle size is critical: the nanosize class (< 200 nm) of PM (nPM) from engineered materials had the greatest toxicity and uptake by vascular and brain cells (Nel et al. 2006; Oberdörster et al. 2005). Similarly, ambient urban nPM shows enhanced biological effects. In rodent atherosclerosis models, inhaled nPM was more active than are larger particles (Araujo et al. 2008). Moreover, aqueous suspensions of nPM were cytotoxic to murine macrophages, possibly by generating reactive oxygen species abiotically (Xia et al. 2006).

Inhaled nPM can act on the brain directly and indirectly. Direct entry of inhaled nPM may occur via transport by olfactory neurons from the nasal mucosa, as modeled by the uptake of inhaled 37-nm carbon-13 particles into the brain (Oberdörster et al. 2004). Another possible route is through peripheral monocytes, which regularly enter the pool of brain microglia, with enhanced entry during peripheral inflammation (D’Mello et al. 2009). Furthermore, systemic IL-1 and other inflammatory mediators induced by nPM can be transported across the blood–brain barrier (Banks et al. 2002–2003; Nguyen et al. 2002; Plotkin et al. 2000). However, neuronal responses to inhaled nPM have not been reported.

We therefore studied neuronal cell responses to nPM *in vivo* after chronic exposure for 10 weeks, using a new technique of reaerosolized nPM collected from urban freeway air for 30 days. We directly exposed *in vitro* brain cells to aqueous nPM suspensions. Additionally, we tested the possibility that nPM could be neurotoxic through glutamatergic mechanisms, particularly *N*-methyl-d-aspartic acid (NMDA) receptors, which are implicated in hypoxic-ischemic neuronal damage and excitotoxic cell death (Hardingham and Bading 2010; Lynch and Guttmann 2002; Mizuno et al. 2008). Lastly, we used *in vitro* models with cultured neurons and glia to evaluate direct and indirect actions of nPM on developing neurons.

## Materials and Methods

*nPM collection and transfer into aqueous suspension.* We collected nPM with a high-volume ultrafine particle (HVUP) sampler (Misra et al. 2002) at 400 L/min flow in Los Angeles City near the CA-110 Freeway. These aerosols represent a mix of fresh ambient PM mostly from vehicular traffic nearby this freeway (Ning et al. 2007). The HVUP sampler consists of an ultrafine particle slit impactor, followed by an after-filter holder. The nPM (diameter < 200 nm) was collected on pretreated Teflon filters (20 × 25.4 cm, polytetrafluoroethylene, 2 μm pore; Pall Life Sciences, Covina, CA). We transferred the collected nPM into aqueous suspension by 30 min soaking of nPM-loaded filters in Milli-Q deionized water (resistivity, 18.2 MW; total organic compounds < 10 ppb; particle free; bacteria levels < 1 endotoxin units/mL; endotoxin-free glass vials), followed by vortexing (5 min) and sonication (30 min). As a control for *in vitro* experiments with resuspended nPM, fresh sterile filters were sham extracted. Aqueous nPM suspensions were pooled and frozen as a stock at –20°C, which retains chemical stability for ≥ 3 months (Li N et al. 2003; Li R et al. 2009). For *in vitro* experiments, nPM suspensions were diluted in culture medium, vortexed, and added directly to cultures.

*Animals and exposure conditions.* The nPM suspensions were reaerosolized by a VORTRAN nebulizer (Vortran Medical Technology 1 Inc., Sacramento, CA) using compressed particle-free filtered air [see Supplemental Material, Figure S1 (doi:10.​1289/ehp.1002973)]. Particles were diffusion dried by passing through silica gel; static charges were removed by passing over polonium-210 neutralizers. Particle sizes and concentrations were continuously monitored during exposure at 0.3 L/min by a scanning mobility particle sizer (SMPS model 3080; TSI Inc., Shoreview, MN). The nPM mass concentration was determined by pre- and postweighing the filters under controlled temperature and relative humidity. Inorganic ions [ammonium (NH_4_^+^), nitrate (NO_3_^–^), sulfate (SO_4_^2–^)] were analyzed by ion chromatography. PM-bound metals and trace elements were assayed by magnetic-sector inductively coupled plasma mass spectroscopy. Water-soluble organic carbon was assayed by a GE-Sievers liquid analyzer (GE-Sievers, Boulder, CO). Analytic details for nPM-bound species are given by Li R et al. (2009). Samples of the reaerosolized nPM were collected on parallel Teflon filters for electron paramagnetic resonance (EPR) analysis.

Mice (C57BL/6J males, 3 months of age) were maintained under standard conditions with *ad libitum* Purina Lab Chow (Newco Purina, Rancho Cucamonga, CA) and sterile water. Just before nPM exposure, mice were transferred from home cages to exposure chambers that allowed free movement. Temperature and airflow were controlled for adequate ventilation and to minimize buildup of animal-generated contaminants [skin dander, carbon dioxide (CO_2_), ammonia]. Reaerosolized nPM or ambient air (control) was delivered to the sealed exposure chambers for 5 hr/day, 3 days/week, for 10 weeks. Mice did not lose weight or show signs of respiratory distress. Mice were euthanized after isoflurane anesthesia, and tissue was collected and stored at –80°C. All rodents were treated humanely and with regard for alleviation of suffering; all procedures were approved by the University of Southern California Institutional Animal Care and Use Committee.

*EPR spectroscopy of nPM.* The reaerosolized nPM was collected on filters (described above), which were inserted directly in the EPR quartz tube (Bruker EPR spectrometer; Bruker, Rheinstetten, Germany); spectra were measured at 22°C. The g-value was determined following calibration of the EPR instrument using DPPH (2,2-diphenyl-1-picrylhydrazyl) as a standard. The EPR signal for DPPH was measured and the corresponding g-value was calculated. The difference from the known g-value of 2.0036 for DPPH was then used to adjust the observed g-value for the sample.

*Cell culture and nPM exposure.* Hippocampal slices from postnatal day 10–12 rats were cultured 2 weeks in a humidified incubator (35°C/5% CO_2_) (Jourdi et al. 2005) with nPM suspensions added for 24–72 hr of exposure. Primary neurons from embryonic day 18 rat cerebral cortex were plated at 20,000 neurons/cm^2^ on cover slips coated with poly-d-lysine/laminin and cultured in Dulbecco’s modified Eagle’s medium (DMEM) supplemented with B27, at 37°C in 5% CO_2_ atmosphere (Rozovsky et al. 2005). Primary glial cultures from cerebral cortex of neonatal day 3 rats (F344) were plated at 200,000 cells/cm^2^ in DMEM/F12 medium supplemented with 10% fetal bovine serum and 1% l-glutamine and incubated as described above (Rozovsky et al. 1998). For conditioned medium experiments, glial cultures were treated with 10 mg nPM/mL; after 24 hr, media were transferred by pipette to neuron cultures.

*Neurite outgrowth and toxicity assays.* After treatments, neurons were fixed in 4% paraformaldehyde and immunostained with anti–β-III-tubulin (1:1,000, rabbit; Sigma Chemical Co., St. Louis, MO); F-actin was stained by rhodamine phalloidin (1:40; Molecular Probes, Carlsbad, CA). A neurite was defined as a process extending from the cell soma of the neuron that was immunopositive for both β-III-tubulin (green) and F-actin (red). The length of neurites was measured using NeuronJ software (Meijering et al. 2004). Growth cones were defined by the presence of actin-rich filopodia and lamellipodia (Kapfhammer et al. 2007). Collapsed growth cones were defined as actin-rich neuritic endings in which filopodia and lamellipodia were indistinguishable. In neurite outgrowth and growth cone collapse assays, individual neurons were selected from two cover slips per condition; *n* is the total number of neurons analyzed per treatment. Cytotoxicity in slice cultures was assayed by lactate dehydrogenase (LDH) release to media and by cellular uptake of propidium iodide (PI) (Jourdi et al. 2005). Neuronal viability was assayed by Live/Dead Cytotoxicity Kit (Invitrogen, Carlsbad, CA) by computer-assisted image analysis of fluorescent images. Mitochondrial reductase was assayed by 3-(4,5-dimethylthiazol-2-yl)-2,5-diphenyltetrazolium bromide (MTT) at 585 nm in undifferentiated PC12 cells (Mosmann 1983). For viability assays, *n* is the total number of hippocampal slices analyzed (LDH release and PI uptake) or the total number of cell culture wells analyzed per condition.

*Immunoblotting.* Mouse hippocampi were homogenized using a glass homogenizer in cold lysis buffer as described by Jourdi et al. (2005). After sample preparation, 20 μg protein was electrophoresed on 10% sodium dodecyl sulfate polyacrylamide gels, followed by transfer to polyvinylidene fluoride (PVDF) membranes. The PVDF membranes were blocked with 5% bovine serum albumin for 1 hr and probed with primary antibodies overnight at 4°C: anti-GluA1 (glutamate receptor subunit 1; 1:3,000, rabbit; Abcam, Cambridge, MA), anti-GluA2 (1:2,000, rabbit; Millipore, Billerica, MA), anti-PSD95 (1:1,000, mouse; Abcam), anti-synaptophysin (1:5,000, mouse; Stressgene; Enzo, Plymouth Meeting, PA), and anti-β-III tubulin (loading control; 1:15,000, rabbit; Sigma), followed by incubation with secondary antibodies (1:10,000) conjugated with IRDye 680 (rabbit, LI-COR Biosciences, Lincoln, NE) and IRDye 800 (mouse, LI-COR). Immunofluorescence was detected by infrared imaging (Odyssey, LI-COR).

*Quantitative polymerase chain reaction (qPCR).* Total cellular RNA was extracted from cerebral cortex of nPM-exposed mice and rat primary glia (Tri Reagent; Sigma), and cDNA (2 μg RNA; Superscript III kit; Invitrogen) was analyzed by qPCR, with primers appropriate for mouse (*in vivo*) or rat (*in vitro*). Genes examined by qPCR were *CD14*, *CD68*, *CD11b*, *CD11c*, *GFAP (*glial fibrillary acidic protein), *IFN-*γ (interferon-γ), *IL-1*α, *IL-1*, *IL-6*, and *TNF*α. Data were normalized to β-actin.

*Statistical analysis.* Data are expressed as mean ± SE. The numbers of individual measurements (*n*) are described above and listed in the figure legends. Single and multiple comparisons used Student’s *t*-test (unpaired) and one-way analysis of variance (ANOVA)/Tukey’s honestly significant difference, with statistical significance defined as *p* < 0.05.

## Results

**Figure 1:**
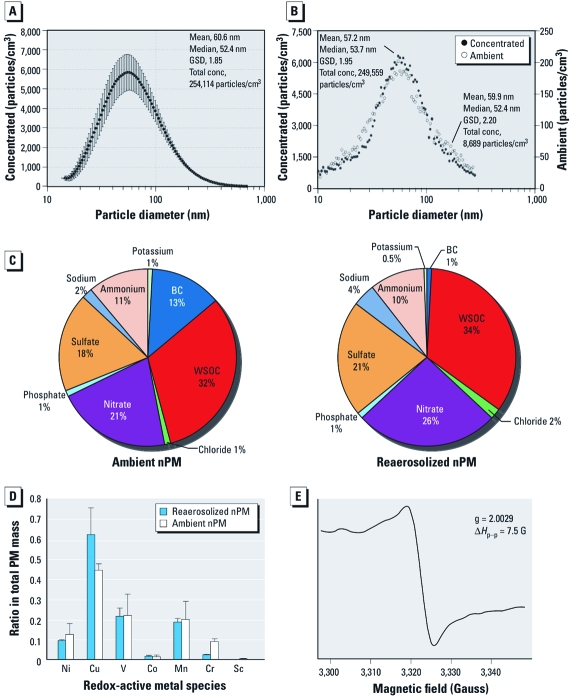
Particle size distributions for ambient and reaerosolized nPM. Abbreviations: Co, cobalt; conc, concentration; Cr, chromium; Cu, copper, GSD, geometric SD; Mn, manganese; Ni, nickel; Sc, scandium; V, vanadium. (*A*) Average aerosol size distributions of reaerosolized nPM collected at the downtown Los Angeles site and used in this study. (*B*) Average ambient and concentrated reaerosolized nPM size distributions from the same downtown Los Angeles site. (*C*) Ambient (left) and reaerosolized (right) nPM bulk chemical composition. The mass ratios of water-soluble organic carbon (WSOC) and inorganic ions between the two aerosols are similar, except for the lower percentage of BC in reaerosolized nPM. (*D*) Redox-active metals in ambient and reaerosolized nPM. (*E*) The EPR signal of nPM from urban Los Angeles vehicular traffic collected 30 days before.

*nPM collection and characterization.* Urban ambient nPM was collected on filters for 30 days, transferred to sterile aqueous suspension, and reaerosolized for inhalation [Supplemental Material, Figure S1 (doi:10.1289/ehp.1002973)]. The reaerosolized nPM particle number and mass concentrations were 2.54 ± 0.47 × 10^5^ particles/cm^3^  (mean ± SE) and 468 ± 25 μg/m^3^, respectively; most (> 95%) particles were < 180 nm in diameter (Figure 1). The size distribution of reaerosolized nPM (Figure 1A) was comparable to typical ambient aerosols of fractioned nPM at peak traffic times at this location (Figure 1B). The chemical composition was also similar for NH_4_^+^, NO_3_^–^, SO_4_^2–^, water-soluble organics (Figure 1C), and water-soluble redox active metals (Figure 1D). However, transfer of BC (Figure 1C) and of the most hydrophobic organic components (PAHs, organic acids) was incomplete (see Supplemental Material, Figure S2).

We observed definitive free radical signals on EPR spectroscopy in a sample of reaerosolized nPM collected during mouse exposure (Figure 1E). The g-value of 2.0029 with a peak-to-peak difference (Δ*H*_p–p_) of 7.5 Gauss is characteristic of stable carbon-based free radicals (Dellinger et al. 2001, 2007). These long-lived free radicals had persisted in frozen aqueous suspension for at least 30 days since the last day of collection from ambient air.

**Figure 2:**
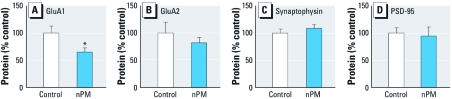
Chronic exposure of mice to nPM caused a decrease of GluA1 but no change in GluA2, synaptophysin, or PSD95 in hippocampus *in vivo*, shown by immunoblot analysis of whole hippocampal lysates of nPM- and control air-exposed mice. (*A*) GluA1 levels were reduced 36% in hippocampal lysates from nPM versus control (*n* = 7 hippocampi per group). No significant changes were seen in GluA2 (*B*) or presynaptic [synaptophysin (*C*)], or postsynaptic [PSD95 (*D*)] marker proteins. *p = 0.04 by *t*-test.

*Chronic nPM mouse exposure.* Male mice exposed to reaerosolized nPM for 10 weeks (3 days/week, 5 hr/day; 150 hr total), showed neuronal responses in the hippocampus, a brain region that mediates learning and memory. Neuronal glutamate receptor subunits in hippocampus responded selectively to nPM inhalation: GluA1 protein levels were decreased by 35% (Figure 2A), whereas GluA2 was not significantly altered (Figure 2B). We also observed no significant change in levels of the major synaptic proteins synaptophysin (presynaptic) and PSD95 (postsynaptic) (Figure 2C,D), consistent with the normal histological appearance of hippocampal neuron layers (data not shown).

**Figure 3:**
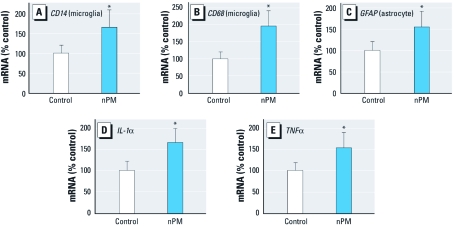
*In vivo* exposure of mice to chronic nPM induced inflammatory responses, shown by qPCR analysis of cerebral cortex mRNA of mice exposed to reaerosolized nPM at levels equivalent to peak vehicular traffic for 10 weeks, 3 days/week, 5 hr/day, as described in “Materials and Methods.” In cerebral cortex, nPM increased mRNA levels of innate immune receptor *CD14 *by 75% (*A*); the microglial marker *CD68 *by 100% (*B*); the astrocytic marker *GFAP* by 50% (*C*); and the two proinflammatory cytokine mRNAs *IL-1*α (*D*) and *TNF*α (*E*) (*n* = 7 cortices per group). *p < 0.05, compared with control air-exposed mice.

Glial activation was indicated by increased mRNA of the microglial markers *CD14* (innate immune receptor) (Figure 3A) and *CD68* (macrosialin) (Figure 3B), and of astrocytic *GFAP* (Figure 3C). Two proinflammatory cytokine mRNAs increased, *IL-1*α (Figure 3D) and *TNF*α (Figure 3E), confirming previous immunohistochemical observations in mice exposed to ambient nPM from the same urban site [see Supplemental Material, Table S1 (doi:10.1289/ehp.1002973)]. *IL-1*β mRNA was also increased, whereas *IL-6*, *IFN-*γ, *CD11b*, and *CD11c* mRNAs were not significantly changed (data not shown).

**Figure 4:**
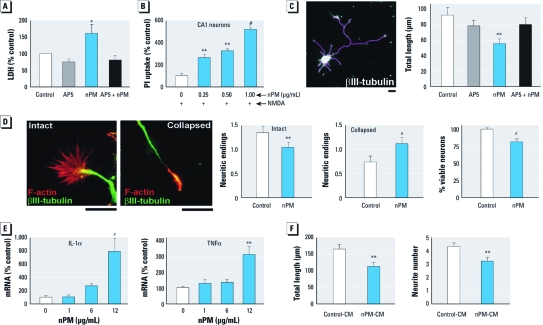
Aqueous suspensions of nPM caused *in vitro* glutamatergic neurotoxicity and inflammatory responses. (*A*) In cultured rat hippocampal slices, nPM caused glutamate-receptor–dependent neuronal damage (LDH release into culture media), which was blocked by the NMDA receptor antagonist AP5 (1 μg/mL nPM, 48 hr, + 50 μM AP5; *n* = 16 hippocampal slices per treatment). (*B*) nPM increased NMDA-induced neurotoxicity (10 μM NMDA, 24 hr treatment with 24 hr recovery). PI uptake was measured as a percentage of NMDA-treated slices (*n* = 5 slices per treatment). (*C*) In primary E18 neuronal cultures, nPM treatment reduced neurite outgrowth; left, representative image used to measure the length of neurites (bar = 20 μm); right, exposure to nPM (2 μg/mL, 48 hr) decreased the total length of neurites by 40% (*n* = 50 neurons per treatment). The inhibition of neurite extension was rescued by the glutamate receptor antagonist AP5 (50 μM). (*D*) In primary neuron cultures, nPM caused > 50% of growth cones to collapse (*n* = 90 neurons per treatment). Intact growth cones retain actin-rich (red) neuritic endings (filopodia and lamellipodia (representative images; bars = 20 μm). nPM (2 μg/mL, 48 hr) decreased neuronal viability by 20% (*n* = 10 culture wells per treatment). (*E*) In mixed glial cultures, 24-hr treatment with nPM caused dose-dependent increase in mRNA levels of inflammatory cytokines IL-1α and TNFα (*n* = 6 glia cultures per treatment). (*F*) Conditioned medium (CM) from nPM-treated mixed glia (10 μg/mL nPM, 24 hr) inhibited neurite outgrowth by 30% and decreased the number of neurites by 25% (*n* = 80 neurons per treatment). *p < 0.05, **p < 0.01; ^#^*p* < 0.001, by ANOVA (*A–C, E*) or *t*-test (*D,F*), compared with control air-exposed mice

*In vitro nPM brain cell exposure.* We evaluated direct effects of nPM suspensions on developing rat hippocampal slice and embryonic neuron cultures. Initial studies calibrated the range of nPM effects using the neuronlike PC12 cell line. By the MTT assay, nPM induced oxidative stress with dose responses from 1 to 20 μg/mL and EC_50_ (50% effective concentration) of 10 μg/mL [see Supplemental Material, Figure S3 (doi:10.1289/ehp.​1002973)]. Because of neuronal changes in glutamate receptors *in vivo* (Figure 2A), we examined hippocampal slice cultures from neonatal rats for nPM cytotoxicity in combination with glutamatergic drugs (Wang and Qin 2010). Release of LDH was increased by nPM at 1 μg/mL during a 48-hr exposure and was blocked by (2*R*)-amino-5-phosphonopentanoate (AP5; NMDA receptor antagonist) (Figure 4A). In the presence of NMDA (a glutamatergic agonist), nPM was also neurotoxic (based on PI uptake in CA1 pyramidal neurons) (Figure 4B). We chose this NMDA dose to target CA1 neurons (effects on CA3 and dentate granule neurons are not shown).

Neurite outgrowth of primary E18 neuronal cultures was inhibited by nPM (2 μg/mL) and rescued by AP5 (Figure 4C), consistent with neurotoxicity assays of cultured slices (Figure 4A). The presence of nPM decreased the number of intact growth cones by 25% and decreased neuron viability by 20% (Figure 4D).

Glia also responded to nPM but at ≥ 5-fold higher concentrations than neuronal responses. In mixed glial cultures (astrocytes and microglia, 3:1), 12 μg/mL nPM increased *IL-1*α and *TNF*α mRNA (Figure 4E). *IL-1*β and *IL-6* mRNAs were also increased, whereas *IFN-*γ mRNA was not significantly changed (data not shown). Indirect effects of nPM on neurons through glial secretions were examined by measuring neurite outgrowth in conditioned medium from nPM-treated glia, which showed an inhibition of neurite outgrowth by 30% and a reduction of neurite number by 25% (Figure 4F).

## Discussion

Neuronal glutamatergic functions responded to urban nPM *in vivo* after chronic inhalation by adult mice for 10 weeks and *in vitro* after 48-hr exposures of neonatal hippocampal slice or embryonic neuron cultures. Inflammatory and oxidative mechanisms were also implicated. The adverse effects of nPM on embryonic neurite outgrowth may be a model for developmental impairments.

We used a novel technology for experimental exposure to airborne PM. Ambient freeway nPM was collected on filters for 1 month and then transferred into an aqueous suspension for reaerosolization at the time of rodent exposure. This approach eliminates the expensive, space-limited, on-site collection facilities for exposing animals to ambient air and has the advantage of collecting nPM over long periods, which averages daily variations. The frozen nPM aqueous suspension can be stored for reaerosolization or direct addition to cultured brain cells. The composition of reaerosolized nPM versus ambient samples was similar except for its lower content of BC and of the most hydrophobic PAHs and other organic components. Nonetheless, despite the reduced nPM content of some of these components, the glial responses to reaerosolized nPM closely approximated those in prior studies of inhaled ambient nPM from this freeway site [see Supplemental Material, Table S1 (doi:10.1289/ehp.1002973)].

We observed two new cellular reactions to nPM in rodent microglia and neurons: chronic nPM inhalation induced the microglial-monocytic markers CD14 (innate immune receptor) and CD68/macrosialin (lysosomal-associated membrane protein) and reduced neuronal GluA1 receptor protein levels. Thus, the microglial activation observed in Mexico City brains from humans (Calderón-Garcidueñas et al. 2008) and canines (Calderón-Garcidueñas et al. 2003) might have resulted from the nPM present in the complex mixture of urban air pollutants. Although the GluA1 receptor is associated with excitotoxic pathways (e.g., Mizuno et al. 2008), we found no evidence for gross neurodegeneration by the normal appearance of hippocampal neuron layers or by the unchanged levels of two synaptic proteins, synaptophysin and PSD95.

*In vitro* models with developing brain cells indicate neurotoxicity of aqueous suspensions of nPM. In cultured neonatal hippocampal slices exposed to nPM for 48 hr, CA1 neurons showed increased uptake of PI, an indicator of cell death. We examined CA1 neurons because of their vulnerability to damage from cerebral ischemia and Alzheimer disease (Simonian and Hyman 1995). Because of likely glia–neuron interactions in cultured slices, we also examined the effects of nPM on cultured embryonic neurons. Direct neuronal effects of nPM included decreased neurite extension, which again was attenuated by AP5. These responses are consistent with the role of glutamatergic NMDA receptor signaling during neurite outgrowth in developing neurons (Ben Fredj et al. 2010; Martel et al. 2009; Mattson et al. 1988).

These *in vitro* models suggest that nPM has both direct neurotoxicity through glutamatergic mechanisms and indirect neurotoxicity through glial secretions. Indirect effects on neurons from nPM-induced glial secretions are indicated by the inhibition of neurite outgrowth in conditioned medium from nPM-treated glia. TNFα, which is induced in nPM-treated glia (Figure 4E) and can inhibit neurite outgrowth (Kato et al. 2010; Neumann et al. 2002), is a candidate for further study in glia–neuron synergies of nPM neurotoxicity. Another mechanism is suggested by the rapid induction of hydrogen peroxide (H_2_O_2_) production by nPM in macrophages within 1 hr (Xia et al. 2006). Glutamatergic NMDA receptors are sensitive to H_2_O_2_ (Bodhinathan et al. 2010; Yang et al. 2010). It will be difficult to evaluate the relative roles of indirect (systemic) influences from cytokines and circulating macrophages from direct effects of nPM transported into the brain by olfactory neurons (see introductory remarks). Nonetheless, further *in vivo* studies are needed to identify the range of neuronal systems responsive to inhaled nPM.

The biochemical basis for nPM neurotoxicity could involve free radicals, including reactive oxygen species. Filter-collected nPM from reaerosolized nPM aqueous suspensions in the present studies has an EPR signal characteristic of stable carbon-based free radicals (Dellinger et al. 2001, 2007). This EPR signal is provisionally generated from the nPM-associated graphitelike BC assayed in the reaerosolized nPM sample (Figure 1C). We detected these signals in nPM collected at least 30 days before measurement. Stable free radicals in nPM are consistent with the generation of singlet oxygen and other ROS *in vitro* (Nel et al. 2006; Oberdörster et al. 2005). In fact, nPM suspensions from this Los Angeles site generated ROS under abiotic conditions (Xia et al. 2006).

The evident neurotoxicity of nPM suggests links between urban air pollution and brain health across the life span. The impairment of neurite outgrowth by nPM is consistent with epidemiological evidence of developmental effects of air pollution in children (Perera et al. 2009; Suglia et al. 2008), which include an association with autism (Volk et al. 2010). The interaction of nPM with glutamatergic (NMDA) excitotoxicity suggests that cerebral ischemia, which also involves glutamatergic excitotoxicity, could be exacerbated by nPM. The increased risk of cardiovascular events during surges of air pollution (“inhaling a heart attack”) (Poloniecki et al. 1997) can reduce cerebral blood flow, with additional risks to the brain. For example, nPM effects could compound neuronal damage in CA1 neurons from cerebral ischemia during stroke or transient ischemia. Moreover, the association of PM with accelerated carotid atherosclerosis (increased carotid artery intima-media thickness) (Künzli et al. 2010) has direct relevance to cognitive aging because of the links between cardiovascular disease and age-related cognitive decline (Haan et al. 1999; Qiu et al. 2006). Urban airborne pollutants may also be a risk factor in Alzheimer disease, as suggested by Calderón-Garcidueñas et al. (2008), who observed that brain amyloid-β peptide deposits in postmortem samples from young adult humans exposed to urban pollution were more prevalent in carriers of apolipoprotein E4, a genotype at high risk for Alzheimer disease.

An important unknown is the actual brain accumulation of inhaled PM, which may be transported inside the brain by olfactory neurons from the nasal mucosa (Oberdörster et al. 2004). Additionally, because of its small size, nPM is not efficiently cleared by mucosal surfaces or alveolar macrophages and is distributed throughout the body, including the brain (Oberdörster et al. 2004, 2005).

Particle size distributions are important for public health. Notably, the smallest PM size for which the U.S. Environmental Protection Agency (EPA) provides quantitative risk assessment (U.S. EPA 2009) is the “fine” PM, having and aerodynamic diameter < 2.5 μm (PM_2.5_). Although the term “ultrafine” particle has been used for nPM, these designations do not define a specific size range (Sioutas et al. 2005) and do not reflect their similarity in size or properties to spherical engineered nanoparticles or other types of nanosized particles (Oberdörster et al. 2005).

Lastly, we note the health problems associated with the global growth of airborne pollutants and the continuing increase of urban vehicular traffic and power generation from fossil fuel. The National Research Council report *America’s Climate Choices: Panel on Advancing the Science of Climate Change* (National Research Council 2010) briefly identified increasing air pollution in urban health but did not discuss endangerment of the brain. Despite the apparent greater toxicity of nPM relative to larger PM, its overall health effects are not well defined by the U.S. EPA (2009). The present findings with portable nPM should facilitate studies on the neurotoxicity and associated risks of nPM to brain development and aging.

## Conclusions

Responses of rodent neurons to airborne nanosized particles from vehicular traffic provide a new model to study associations of urban air pollution to cognitive impairment and neuropathology. Our findings support glutamatergic mechanisms of neuronal response to nPM that may be relevant to interactions of air pollution with cerebral ischemia.

## Supplemental Material

(312 KB) PDF
