# Supplementary material for: Glutamatergic Neurons in Rodent Models Respond to Nanoscale Particulate Urban Air Pollutants in Vivo and in Vitro
Source: Environ Health Perspect. 2011 Apr 7;119(7):1003–9. doi: 10.1289/ehp.1002973 (PMC3222976; doi:10.1289/ehp.1002973)
Supplement: (312 KB) PDF [file ehp.1002973.s001.pdf]

Supplemental Material

Glutamatergic Neurons in Rodent Models Respond to Nanoscale Particulate Urban Air Pollutants *In Vivo* and *In Vitro*

Todd E Morgan[1], David A Davis[1], Nahoko Iwata[1], Jeremy A Tanner[2], David Snyder[2], Zhi Ning[3], Winnie Kam[3], Yu-Tien Hsu[2,4], Jeremy W Winkler[2,5], Jiu-Chiuan Chen[6], Nicos A Petasis[2,5], Michel Baudry[2,4], Constantinos Sioutas[3], and Caleb E Finch[1,4]

[1]Davis School of Gerontology, [2]USC College, [3]Viterbi School of Engineering, [4]Dept. of Neurobiology, [5]Dept. of Chemistry, [6]Keck School of Medicine, University of Southern California, Los Angeles, CA, 90089

Table of Contents:

Figure S1. nPM exposure apparatus and flow chart.

Figure S2. Comparison of the categorized organic compound mass ratio in ambient nPM and re-aerosolized nPM.

Figure S3. nPM decreases mitochondrial function in PC12 cells.

Table S1. Brain responses to ambient or re-aerosolized nPM.

Figure S1:

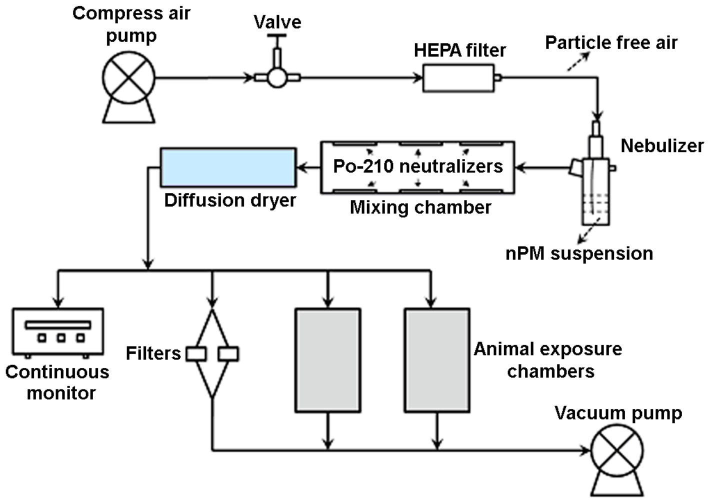

Supplemental Material, Figure S1. nPM exposure apparatus and flow chart.

Figure S2:

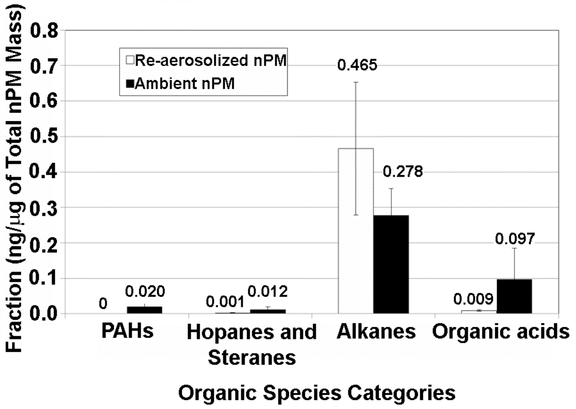

Supplemental Material, Figure S2. Comparison of the categorized organic compound mass ratio in ambient nPM and re-aerosolized nPM.

Figure S3:

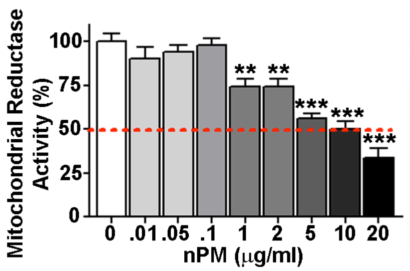

Supplemental Material, Figure S3. nPM decreases mitochondrial function in PC12 cells. Undifferentiated PC12 cells were treated with increasing concentrations of nPM for 48 hrs. Exposure to nPM decreases mitochondrial reductase activity (MTT assay) in a dose-dependent manner with a EC_{50} = 9.37μg/ml (ANOVA; n=48 culture wells per dose; **, P<0.01; ***, P<0.001).

Table S1: Brain responses to ambient[a] or re-aerosolized[b] nPM

	Campbell *et al* 2005[a]	Kleinman *et al* 2008[a]	Campbell *et al* 2009[a]	Morgan et al[b]
	BALB/C; 6 wk exposure	APOE -/-; 6 wk exposure	APOE-/-; 6 wk exposure	C57BL/6; 12 wk exposure
GFAP	N/A	+50% protein	N/A	+60% mRNA
IL1a	+300% protein	N/A	+190% protein	+70% mRNA
TNFa	+25% protein	N/A	+930% protein	+50% mRNA

N/A = Not available

References:

Campbell A, Araujo JA, Li H, Sioutas C, Kleinman M. 2009. Particulate matter induced enhancement of inflammatory markers in the brains of apolipoprotein E knockout mice. J Nanosci Nanotechnol 9:5099-5104.

Campbell A, Oldham M, Becaria A, Bondy SC, Meacher D, Sioutas C, et al. 2005. Particulate matter in polluted air may increase biomarkers of inflammation in mouse brain. Neurotoxicology 26:133-140.

Kleinman MT, Araujo JA, Nel A, Sioutas C, Campbell A, Cong PQ, et al. 2008. Inhaled ultrafine particulate matter affects CNS inflammatory processes and may act via MAP kinase signaling pathways. Toxicol Lett 178:127-130.
